# The neuroprotective effect of resveratrol on retinal ganglion cells after optic nerve transection

**Published:** 2013-07-25

**Authors:** Seok Hwan Kim, Joo Hyun Park, Yu Jeong Kim, Ki Ho Park

**Affiliations:** 1 Department of Ophthalmology, Seoul National University College of Medicine, Seoul, Korea; 2 Department of Ophthalmology, Seoul National University Boramae Hospital, Seoul, Korea; 3 Department of Ophthalmology, Seoul National University Hospital, Seoul, Korea

## Abstract

**Purpose:**

This study aimed to investigate the neuroprotective effect of resveratrol in an optic nerve transection (ONT) model and to identify the neuroprotective mechanism of resveratrol in retinal ganglion cells (RGCs).

**Methods:**

ONT and retrograde labeling were performed in Sprague-Dawley rats. Various concentrations of resveratrol were injected intravitreally immediately after ONT. The number of labeled RGCs was determined at 1 and 2 weeks after ONT. The effect of resveratrol and sirtinol (a sirtuin 1 inhibitor) co-injection was investigated. RGC-5 cells were cultured and treated with staurosporine to induce differentiation. 3-(4,5-Dimethylthiazol-2-yl)-2,5-diphenyltetrazolium bromide (MTT) assay was performed to evaluate the effect of resveratrol on RGC-5 cell survival under serum-free conditions. RGC-5 cells were cultured with sirtinol to investigate the neuroprotective mechanism of resveratrol.

**Results:**

A dose–response relationship was observed between resveratrol and RGC survival. A single intravitreal injection of resveratrol was neuroprotective in RGCs at 1 week after ONT (p<0.01). Repeated intravitreal injection of resveratrol showed a neuroprotective effect at 2 weeks after ONT (p<0.01). However, co-injection of resveratrol and sirtinol diminished the neuroprotective effect of resveratrol (p<0.05). The neuroprotective effect of resveratrol was observed in RGC-5 cells under serum-free conditions, and sirtinol diminished this neuroprotective effect.

**Conclusions:**

Resveratrol exerts its neuroprotective effect on RGCs via activation of the sirtuin 1 pathway in an ONT model. This finding demonstrates the therapeutic potential of resveratrol in treating optic nerve diseases.

## Introduction

Retinal ganglion cells (RGCs) are the only projecting neurons of the retina. Their axons form the optic nerve and transmit visual information to the brain. Damage to the optic nerve results in retrograde degeneration of RGCs. Degeneration of RGCs in rats is often induced by optic nerve transection (ONT), which leads to a rapid loss of RGCs of up to 90% in 14 days [1,2]. Cell death of axotomized RGCs shows typical characteristics of apoptosis [3]. Although the exact cause of RGC apoptosis is still unknown, several factors are proposed to be involved in RGC death, including activation of the caspase pathway [4,5], growth factor depletion [6], oxidative stress [7], dysregulation of proapoptotic/antiapoptotic signals [8,9], and other factors [10-12]. Several studies have demonstrated that modulation of the associated factors had a neuroprotective effect on RGC survival [7,10-12]. To date, however, no established neuroprotective treatment modality has been found to preserve the RGCs and axons in many optic neuropathies, including glaucoma and traumatic optic neuropathy.

Resveratrol (C_14_H_12_O_3_; *trans*-3,5,4′-trihydroxystilbene) is a natural polyphenol found in grapes, red wine, berries, knotweed, peanuts, and other plants [13]. It has protective effects against cardiovascular diseases, neurodegenerative diseases, aging, and cancer [13-15]. Although the mechanisms by which resveratrol exerts its beneficial effects have not yet been clearly elucidated, several studies have described its antioxidant and anti-inflammatory properties [13]. Recently, resveratrol has been shown to activate sirtuin 1 (SIRT1), which is a member of the sirtuin family of nicotinamide adenine dinucleotide (NAD^+^)-dependent deacetylases [16,17]. Seven sirtuins have been identified in mammals, of which SIRT1 is implicated in many vital processes such as DNA repair, cell survival, gluconeogenesis, muscle cell differentiation, cell cycle regulation, lipid metabolism, and fat mobilization [18]. Resveratrol was found to inhibit neuronal dysfunction in a neuronal and axonal degeneration model through the SIRT1 pathway [19,20], and to exert neuroprotective effects against traumatic injury and ischemic damage in the rat brain [21,22]. Therefore, resveratrol exhibits potency for the treatment of neurodegenerative diseases, particularly optic neuropathies due to RGC loss. The aims of the present study are to investigate the neuroprotective effect of resveratrol in an ONT model and to identify the neuroprotective mechanism of resveratrol in RGCs.

## Methods

### The optic nerve transection model

The use of animals for this study was approved by the Animal Research Committee of the Seoul National University Hospital and was performed in compliance with the Association for Research in Vision and Ophthalmology’s Statement for the Use of Animals in Ophthalmic and Vision Research. Male Sprague-Dawley rats weighing 250 to 300 g were housed, with standard chow and water provided ad libitum. The procedure used to generate the ONT model has been described previously [23]. Briefly, the animals were anesthetized with an intramuscular injection of xylazine (Rompun, Bayer, Germany; 1 mg/kg) and Zoletil (Virbac Animal Health Ltd., Carros, France; 10 mg/kg). The lateral conjunctiva was incised with scissors, and the optic nerve was exposed by blunt dissection. A 2 mm longitudinal incision was made in the optic nerve sheath with a needle knife starting 3 mm behind the eye. A cross-section of the optic nerve was made with a needle knife through the opening of the optic nerve sheath, with care taken to avoid damage to the adjacent blood supply. The conjunctival incision was sutured, and Tobrex ophthalmic ointment (Alcon, Fort Worth, TX) was applied topically. ONT was performed on the left eye of each rat.

### Quantification of retinal ganglion cells

RGC loss in the ONT retinas was evaluated by retrograde labeling with dextran tetramethylrhodamine (DTMR; Molecular Probes, Eugene, OR). After the ONT procedure, DTMR was immediately applied to the proximal cut surface of the optic nerve. Eyes were enucleated and fixed in 4% paraformaldehyde solution at 4 °C for 120 min. The retinas were dissected from the eye cups and prepared as flat mounts, with four radially oriented cuts in each retina. The retinas were then whole mounted on glass slides. The slides were kept in the dark and air dried overnight. The DTMR-labeled RGCs were viewed with a fluorescence microscope, and images were captured with a microscope eyepiece reticle at 400× magnification at 1, 2, and 3 mm from the center of the optic nerve along the centerline of each retinal quadrant. The number of labeled RGCs was manually counted by two investigators in a masked fashion and averaged. The number of labeled cells in the 12 photographs was divided by the area of the region, and the data were pooled to calculate the mean density of labeled cells per square millimeter for each retina.

### Immunohistochemistry

Enucleated eyeballs from deeply anesthetized animals were fixed in 4% paraformaldehyde overnight and embedded in optimum cutting temperature compound. Ten micrometer thick sections were obtained along the vertical meridian through the optic nerve head. After being washed with phosphate buffered solution (PBS; potassium chloride 0.2 g/l, potassium phosphate 0.2 g/l, sodium chloride 8.0 g/l, sodium phosphate 1.15 g/l) containing 0.1% Triton X-100, the sections were incubated with blocking serum solution and then incubated with primary antibody against SIRT1 (Santa Cruz Biotechnology, Santa Cruz, CA) at 4 °C overnight. Antigen–antibody complexes were detected with avidin-biotin-peroxidase (Vectastatin ABC kit, Vector Laboratories, Burlingame, CA). Diaminobenzidine with nickel chloride (diaminobenzidine substrate kit, Vector Laboratories) was used to visualize staining.

### Administration of test agents

Resveratrol (Sigma-Aldrich, St. Louis, MO) was dissolved in PBS and diluted to final concentrations of 4.38, 13.1, 43.8, 131, and 438 uM. Four microliters of resveratrol of each concentration was administered intravitreally with a Hamilton syringe (Hamilton Company, Reno, NV) immediately after the ONT procedure. The same volume of PBS was administered intravitreally in control eyes. Sirtinol (Sigma-Aldrich) was dissolved in PBS and diluted to a final concentration of 100 μM, which is a previously established effective concentration [24]. Four microliters of sirtinol were coadministered intravitreally with resveratrol. The final drug concentration in the eye was estimated to be one-fourteenth the concentration of the solution injected based on the volume injected and the average volume of the vitreal space measured in Sprague-Dawley rat eyes [25]. Concentrations given in the text and figures represent this estimated final dilution.

### Cell culture

RGC-5 cells, a transformed retinal ganglion cell line, were cultured in Dulbecco’s modified Eagle’s medium containing 1 g/l glucose with l-glutamine, supplemented with 10% fetal bovine serum, 100 U/ml penicillin, and 100 μg/ml streptomycin (Sigma-Aldrich). Cells were incubated in a humidified atmosphere of 95% air and 5% CO_2_ at 37 °C. Differentiation of RGC-5 cells was induced by treatment with 1 μM staurosporine in 0.1% dimethyl sulfoxide for 1 h. For immunocytochemistry, cells were fixed with 4% paraformaldehyde for 30 min and washed three times with PBS. After blocking with 5% bovine serum albumin, cells were incubated with primary antibody for Brn-3 (Santa Cruz Biotechnology, Santa Cruz, CA), neurofilament (Dako, Tokyo, Japan), and Thy-1 (Santa Cruz Biotechnology). Characterization of the RGC-5 cell line was performed by immunostaining cultured cells for RGC-specific protein expression. Cells were incubated with fluorescein *isothiocyanate*–conjugated antirabbit or antigoat immunoglobulin G (IgG) antibody (Cappel Research Products, Durham, NC) at 25 °C for 60 min. 4’,6-Diamidino-2-phenylindole was used for nuclear staining. Images were acquired with fluorescence confocal microscopy. Further characterization of the RGC-5 cell line was performed by sequencing of a region of the tRNA-phe gene, as previously described [26]. Briefly, PCR amplification was performed with forward (5′-CTC AAC ATA GCC GTC AAG GC-3′) and reverse primers (5′-ACC AAA CCT TTG TGT TTA TGG G-3′) by first heating to 94 °C for 5 min; 35 cycles of 94 °C for 30 s, 55 °C for 60 s, and 72 °C for 60 s; and a final extension of 72 °C for 5 min. For sequencing, PCR products were sent to the DNA Analysis Core Facility (Seoul, Korea).

### Serum deprivation and test agents

In the serum-deprivation protocol, RGC-5 cells were rinsed three times and incubated in serum-free Dulbecco’s modified Eagle’s medium for 24 and 48 h in a humidified atmosphere of 95% air and 5% CO_2_ at 37 °C. Resveratrol (7.5 μM) was added to the culture medium, and sirtinol (2.5 μM) was used to inhibit SIRT1 activity. After the drugs were combined, they were added simultaneously to the wells.

### Cell viability assay

Cells (1 × 10^4^ per well) were cultured in 100 μl of medium per well for the indicated time. Cell viability was evaluated with 3-(4,5-dimethylthiazol-2-yl)-2,5-diphenyltetrazolium bromide (MTT; Roche Applied Science, Indianapolis, IN) reduction assay. MTT was added to each well at a final concentration of 0.5 mg/mL, and the cells were incubated for an additional 1 h under the same conditions. The medium was then removed from the cultures, and reduced MTT was solubilized by adding 100 μl of dimethyl sulfoxide to each well. The plates were agitated for 15 min, and the optical density of the solubilized formazan product in each well was measured with an automatic microplate reader at a test wavelength of 570 nm and a reference wavelength of 690 nm. Data were presented as percentage of viable cells of the same-day control wells. Each set of data was collected from multiple replicate wells of each experimental group, and at least three independent experiments were performed.

### Statistical analysis

The Mann–Whitney test or one-way analysis of variance (ANOVA) with the post hoc Tukey test was performed for statistical analysis using SPSS 17.0. A value of p<0.05 was considered statistically significant.

## Results

### Analysis of the neuroprotective effect of resveratrol

**Figure 1:**
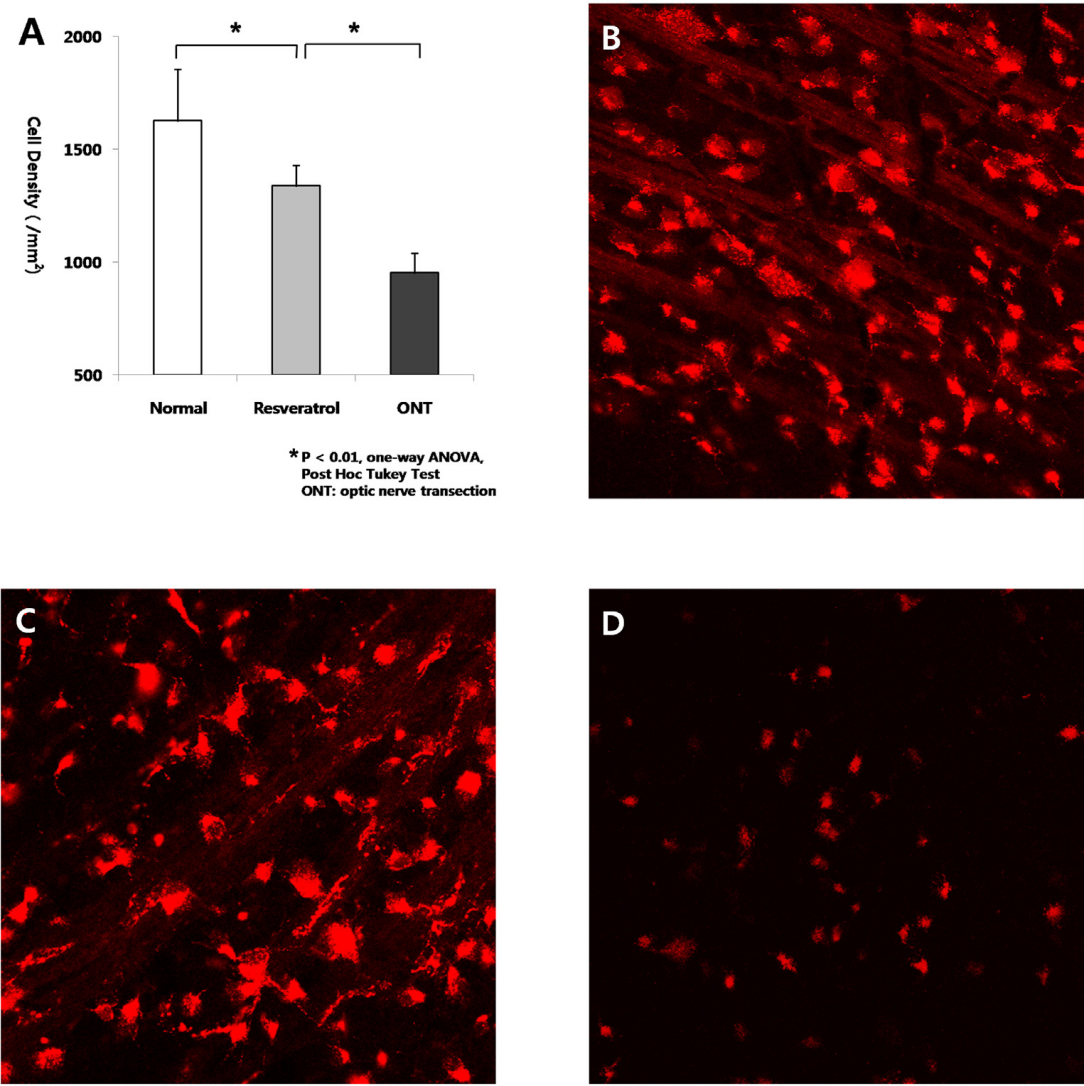
Analysis of neuroprotective effect of resveratrol at 1 week after optic nerve transection. **A**: The numbers of retinal ganglion cells (RGCs) of eyes treated with intravitreal injection of resveratrol (31.3 uM) in the transected rats (**C**) were significantly larger than those with injection of PBS in the transected rats (**D**), but smaller than those with injection of PBS in the nontransected rats (**B**; n=5 for each group, *p<0.01, one-way analysis of variance [ANOVA], post hoc Tukey test, error bars represent standard deviation).

To evaluate the ability of resveratrol to attenuate RGC loss, we intravitreally injected resveratrol (31.3 uM) immediately after ONT. In control eyes, PBS was injected intravitreally after ONT and sham operation. Eyes were enucleated on day 7, and the densities of RGCs were compared among the three groups. Figure 1 shows that eyes with ONT had significantly fewer surviving RGCs at day 7 than normal control eyes. Eyes with ONT treated with a single intravitreal injection of resveratrol (31.3 uM) had significantly more RGCs (n=5; 1337.4±90.5/mm^2^) than eyes with ONT treated with PBS (n=5; 952.2±87.0/mm^2^), but significantly fewer RGCs than eyes without ONT (n=5; 1626.1±227.5/mm^2^; p<0.01, one-way ANOVA, post hoc Turkey test).

### Dose–response relationship between resveratrol and retinal ganglion cell survival

**Figure 2:**
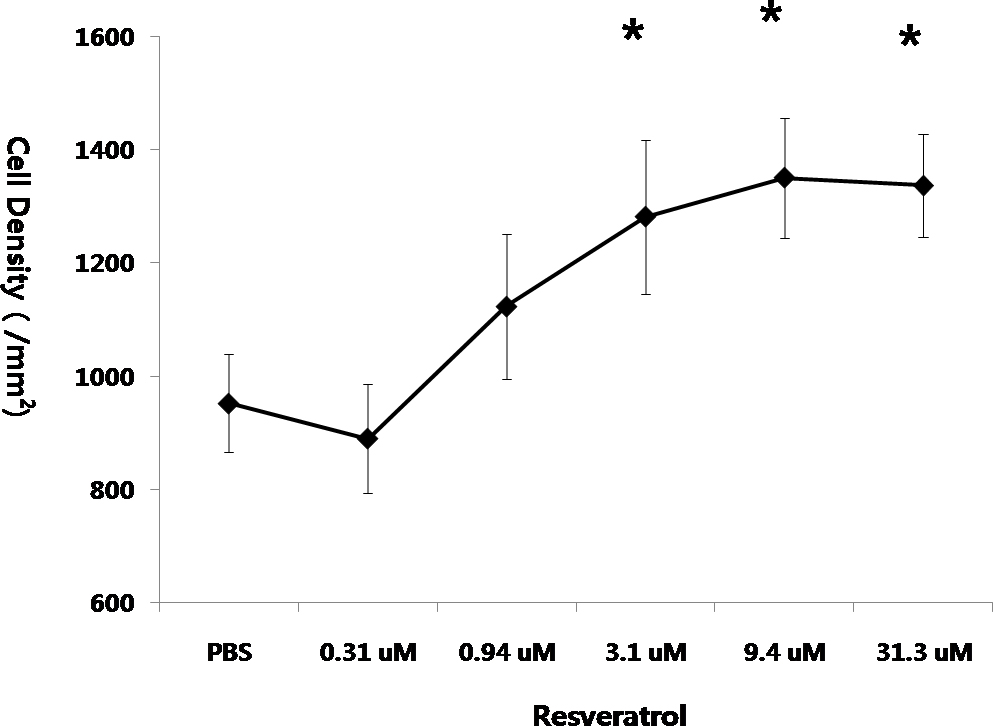
Comparison of the neuroprotective effect of resveratrol according to the different concentrations. In optic nerve transected (ONT) eyes, resveratrol in the concentration of 3.1 uM or more showed statistically significant neuroprotective effect for the retinal ganglion cells (RGCs) compared to the control group (n≥4 for each group, *p<0.01, one-way analysis of variance [ANOVA], post hoc Tukey test, error bars represent standard deviation).

To investigate whether resveratrol attenuated RGC loss in a dose-dependent manner, we intravitreally injected various concentrations of resveratrol (0.31, 0.94, 3.1, 9.4, and 31.3 uM) immediately after ONT. At 1 week after ONT (n≥4), resveratrol had a statistically significant neuroprotective effect on RGCs at a concentration of ≥3.1 uM compared with the control group (p<0.01, one-way ANOVA, post hoc Tukey test; Figure 2). ONT eyes treated with 0.94 uM resveratrol exhibited significantly greater RGC survival than ONT eyes treated with 0.31 uM resveratrol, but had significantly fewer RGCs than ONT eyes treated with ≥9.4 uM resveratrol. There was no difference in the RGC survival among the ONT eyes treated with 3.1, 9.4, and 31.3 uM resveratrol.

### Duration of the neuroprotective effect of resveratrol

**Figure 3:**
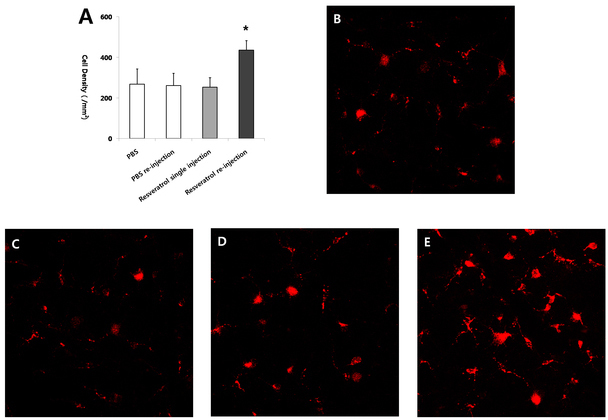
Analysis of neuroprotective effect of resveratrol at 2 week after injection. **A**: The numbers of retinal ganglion cells (RGCs) of eyes treated with resveratrol (31.3 uM) in the transected rats (**D**) were not significantly different from those with injection of PBS in the transected rats (**B**, **C**). However, the numbers of the RGCs of eyes with intravitreal reinjection of resveratrol (**E**) were significantly larger than those with PBS in transected rats (**B**, **C**; n≥5 for each group, *p<0.01, one-way analysis of variance [ANOVA], post hoc Tukey test, error bars represent standard deviation).

To evaluate the duration of the effect of resveratrol, we compared RGC loss at 2 weeks after ONT in eyes treated with a single intravitreal injection of 4 μl of PBS, eyes treated with repeated intravitreal injection of 4 μl of PBS, eyes treated with a single intravitreal injection of resveratrol (31.3 uM; n≥5) after ONT, and eyes treated with repeated intravitreal injection of resveratrol (31.3 uM) at 1 week intervals after ONT. The numbers of RGCs of eyes treated with a single injection of resveratrol (253.3±46.8/mm^2^) did not show a statistically significant difference from those of eyes treated with a single PBS injection (268.1±75.8/mm^2^) or those of eyes treated with repeated PBS injection (260.3±62.3/mm^2^). However, the numbers of RGCs of eyes treated with repeated injection of resveratrol (435.8±46.9/mm^2^) at 2 weeks after ONT were significantly larger than those of the control groups or eyes treated with a single intravitreal injection of resveratrol (p<0.01, one-way ANOVA, post hoc Tukey test), indicating the sustained neuroprotective effect of repeatedly injected resveratrol at 1 week intervals (Figure 3).

### Immunolocalization of sirtuin 1 in the normal rat retina

**Figure 4:**
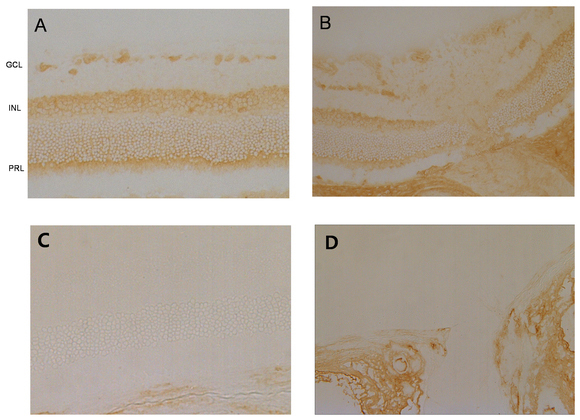
Immunolocalization of sirtuin 1 in the normal rat retina. Immunohistochemical staining showed that sirtuin 1 (SIRT1) was strongly expressed in the retinal ganglion cell (RGC) layer (GCL), inner nuclear layer (INL), outer nuclear layer, and photoreceptor layer (PRL; **A**). SIRT1 was also expressed in the optic nerve axon (**B**). The negative controls showed no detectable staining in retina (**C**, **D**).

Immunohistochemical staining of the rat retinal sections with anti-SIRT1 antibody showed strong expression of SIRT1 in the RGC layer, inner nuclear layer, outer nuclear layer, and photoreceptor layer (Figure 4). Optic nerve axons also showed relatively strong expression of SIRT1. The negative control for immunohistochemistry, in which the SIRT1 primary antibody was omitted, showed no detectable staining.

### Neuroprotection by resveratrol blocked with sirtuin 1 inhibition

**Figure 5:**
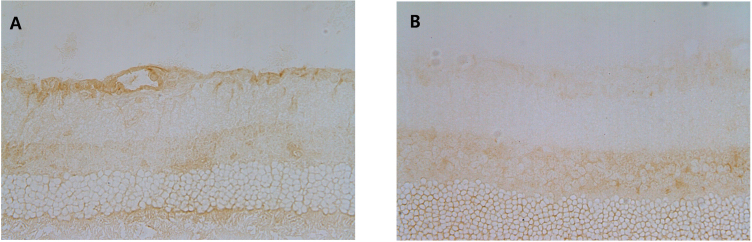
Expression of sirtuin 1 in retinal ganglion cells three days after optic nerve transection in eyes treated with resveratrol injection (**A**) and in eyes treated with co-injections of sirtinol and resveratrol (**B**). The expression of sirtuin 1 (SIRT1) in retinal ganglion cells (RGCs) decreased in eyes treated with co-injections of sirtinol and resveratrol.

**Figure 6:**
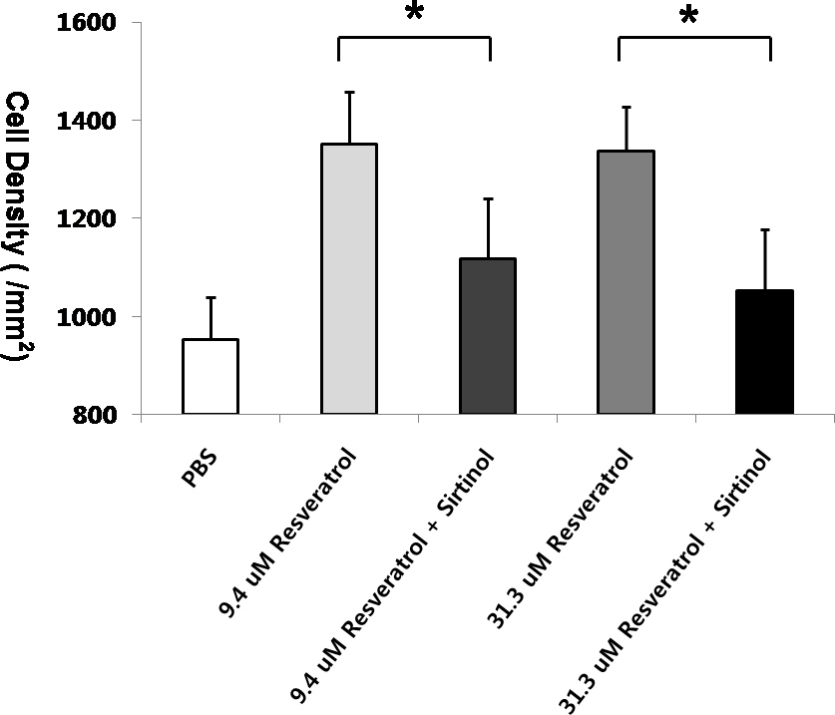
Neuroprotection of resveratrol blocked by sirtinol, a sirtuin 1 inhibitor. The numbers of retinal ganglion cells (RGCs) in eyes co-injected with resveratrol and sirtinol were smaller than injected with resveratrol at 1 week after treatment with concentrations of 9.4 uM and 31.3 uM of resveratrol (n≥5 for each group, *p<0.05, Mann-Whitney test, error bars represent standard deviation).

To confirm that the neuroprotective effect of resveratrol was mediated through the SIRT1 pathway, we blocked SIRT1 activity with a known inhibitor—sirtinol (7.14 μM; Figure 5). We compared the RGC survival of eyes co-injected intravitreally with resveratrol (31.3 uM) and sirtinol to that of eyes injected with resveratrol (31.3 uM) alone (n≥5). RGC survival in eyes treated with both resveratrol and sirtinol was significantly lower than that in eyes treated with resveratrol alone (p<0.05, Mann–Whitney test; Figure 6). Similar results were observed with 9.4 uM resveratrol.

### Neuroprotective effect of resveratrol on RGC-5 cells under serum-deprived conditions

**Figure 7:**
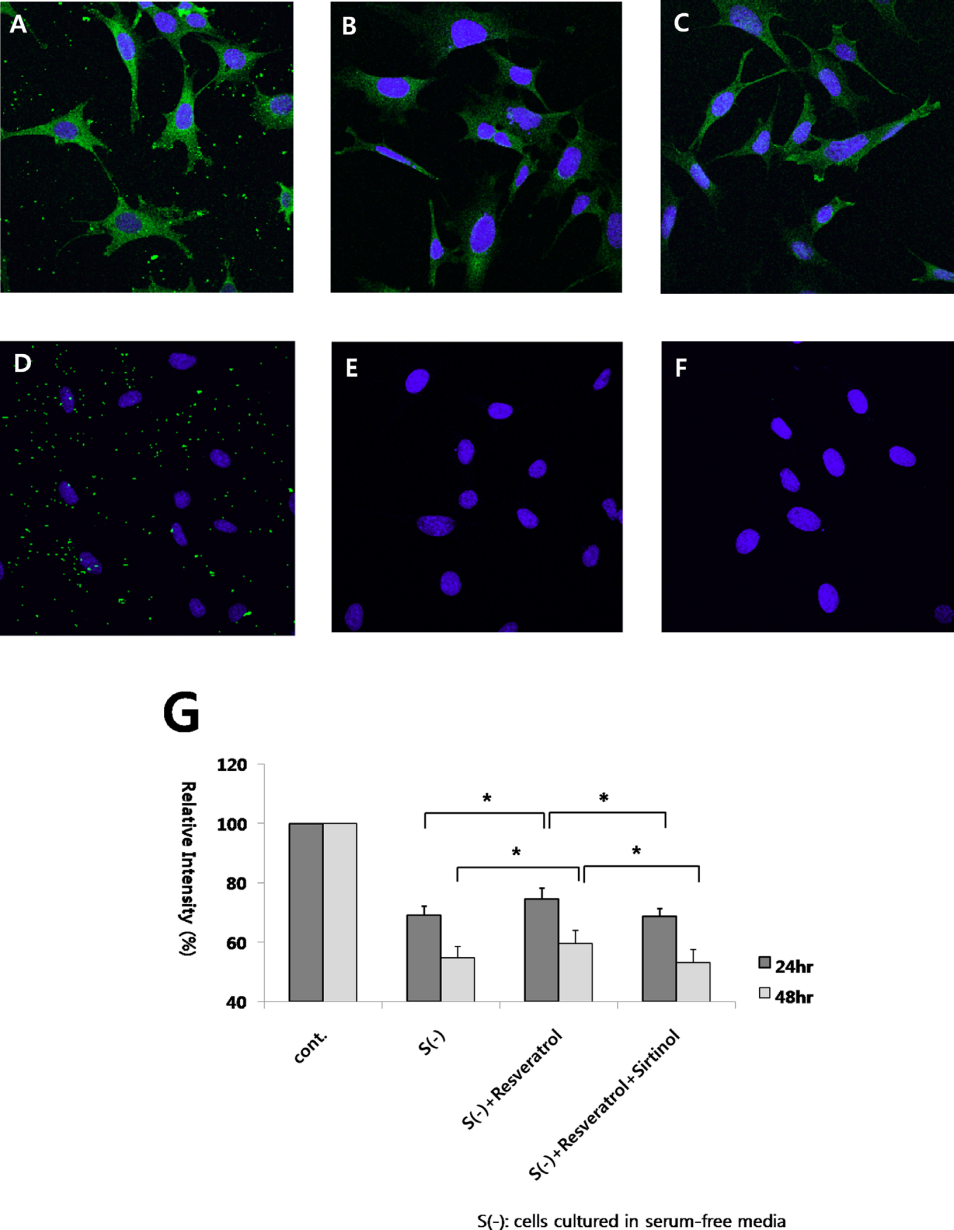
Effect of resveratrol and sirtinol on the survival of RGC-5 cells in serum-free media were shown. The control group had cells cultured in 10% fetal bovine serum; the label S(-) indicated a group with cells cultured in serum-free media. The group with resveratrol addition showed more cell viability than the group with PBS addition in cells cultured in serum-free media. Coaddition of resveratrol and sirtinol resulted in lower cell viabilities than the group with resveratrol only at 24 h and 48 h after addition. (**G**; n ≥ 3 for each group, *p < 0.05, one-way analysis of variance [ANOVA], post hoc Tukey test, error bars represent standard deviation) Immunocytochemistry showed the expression of Brn-3 (**A**), neurofilament (**B**) and Thy-1 (**C**) in RGC-5 cells. The negative controls of Brn-3 (**D**), neurofilament (**E**) and Thy-1 (**F**) were shown, which were stained with only secondary antibody.

We confirmed the expression of several RGC markers in RGC-5 cells, including Brn-3, neurofilament, and Thy-1 (Figure 7). DNA sequencing of a region of the tRNA-phe gene showed that RGC-5 cells shared 99.8% sequence identity with mouse, indicating that this cell line is of mouse origin. We compared the cell viabilities of the three groups (without resveratrol treatment, with resveratrol treatment alone, and with cotreatment of resveratrol and sirtinol) at 24 and 48 h after culture. Using MTT assay, we found a significant increase in cell viability in the resveratrol-treated group compared with the untreated group after 24 and 48 h of incubation in serum-free medium (p<0.05, one-way ANOVA, post hoc Tukey test; Figure 7). We also found a significant decrease in cell viability in the group cotreated with resveratrol and sirtinol compared with the group treated with resveratrol alone at 24 and 48 h after the addition of the compounds (p<0.05, one-way ANOVA, post hoc Tukey test; Figure 7).

## Discussion

Since it was reported in the early 1990s that resveratrol can be found in red wine, many investigators have described its antioxidant, anti-inflammatory, and antitumor activities [13,27]. Resveratrol has also been shown to mimic the effects of caloric restriction and extend the lifespan of several species from yeast to mice by activating SIRT1 [16,17,28]. Recently, resveratrol was reported to have a neuroprotective effect against traumatic brain injury and cerebral ischemic damage in rats [21,22]. Moreover, in the Huntington and Alzheimer disease models, resveratrol inhibited neuronal degeneration via SIRT1 activation [19,29]. Araki et al. reported that cultured neurons treated with resveratrol before axotomy showed a decrease in axonal degeneration, and SIRT1 played a major role in this effect [20]. From these studies, we hypothesized that resveratrol was likely to have a neuroprotective effect on RGC survival after ONT through SIRT1 activation.

In this study, we have addressed the neuroprotective effect of resveratrol on RGCs by combining in vivo and in vitro approaches. Using an ONT model, we found that resveratrol attenuated RGC loss in a dose-dependent manner. We demonstrated the neuroprotective effect of repeated intravitreal injection of resveratrol at 2 weeks after ONT. Using the differentiated RGC-5 cells, which have many of the morphological, postmitotic, electrophysiological, and antigenic properties of mature RGCs [30], we also found that resveratrol increased cell viability under serum-deprived conditions as in the in vitro model of trophic-support interruption. We have investigated the mechanism underlying the neuroprotective effect of resveratrol through in vivo and in vitro experiments. The pharmacological inhibition of SIRT1 with sirtinol significantly reduced the number of surviving RGCs after ONT, indicating that neuroprotection by resveratrol is mediated by the SIRT1 pathway. In addition, using the differentiated RGC-5 cells, we confirmed that resveratrol exerts its neuroprotective effect on RGC survival via the SIRT1 pathway. Altogether, these data showed for the first time the neuroprotective effect of resveratrol and its action mechanism in RGCs after axonal insult.

Resveratrol exerts its neuroprotective effects through diverse pathways. Of these, the SIRT1 (NAD^+^-dependent deacetylase) pathway [31] and the antioxidant pathway [32], which prevent oxidative stress and inhibition of Na^+^K^+^ ATPase activity, can prevent neuronal cell loss. In this study, we identified the neuroprotective effect of resveratrol in an ONT model to occur via the SIRT1 pathway based on the inhibitory effect of sirtinol, a SIRT1 inhibitor, on neuroprotection by resveratrol. However, further studies on the genetic inhibition of SIRT1 should be performed. The specific mechanisms of SIRT1 mediating RGC survival are yet unknown. SIRT1 has been shown to exert its effects by deacetylating transcription factors, such as the tumor suppressor p53 [33,34], the FOXO family [35,36], the transcription factor NF-κβ [37], and Ku70 [38], thereby reducing their ability to trigger apoptosis. Because apoptosis has been shown previously to mediate RGC loss in an ONT model, apoptotic proteins might be candidate downstream targets of SIRT1-mediated RGC neuroprotection. Further studies are warranted to identify specific downstream targets for potential therapeutic intervention.

In this study, a single intravitreal injection of resveratrol provided substantial neuroprotection in the ONT model until 1 week after injection, but did not last for 2 weeks after injection. However, the neuroprotective effect of resveratrol could be sustained by reinjection at 1 week after the first injection. Therefore, resveratrol has a transiently neuroprotective effect, but repeated administration can sustain a prolonged effect on RGCs. This result might provide information regarding the treatment interval of resveratrol administration.

In the present study, we administered resveratrol intravitreally to determine its direct effect on RGCs, as well as to avoid its metabolization. Although resveratrol is known to penetrate the blood–brain barrier, it is rapidly metabolized in the intestine and liver, resulting in low oral bioavailability [39]. A recent study by Li et al. showed that resveratrol pretreatment in mice did not prevent ischemia or reperfusion-induced retinal ganglion cell loss [40]. Because they used oral administration of resveratrol, however, the low oral bioavailability of resveratrol might explain the negative results. Further studies should be performed to evaluate the neuroprotective effect of portal administration of resveratrol.

In conclusion, we showed that resveratrol exerts its neuroprotective effect via activation of the SIRT1 pathway in an optic nerve damage model. This finding demonstrates the therapeutic potential of resveratrol in treating optic nerve diseases.

## References

[1] Mey J, Thanos S (1993). Intravitreal injections of neurotrophic factors support the survival of axotomized retinal ganglion cells in adult rats in vivo.. Brain Res.

[2] Mansour-Robaey S, Clarke DB, Wang YC, Bray GM, Aguayo AJ (1994). Effects of ocular injury and administration of brain-derived neurotrophic factor on survival and regrowth of axotomized retinal ganglion cells.. Proc Natl Acad Sci USA.

[3] Weishaupt JH, Bähr M (2001). Degeneration of axotomized retinal ganglion cells as a model for neuronal apoptosis in the central nervous system - molecular death and survival pathways.. Restor Neurol Neurosci.

[4] Kermer P, Ankerhold R, Klocker N, Krajewski S, Reed JC, Bahr M (2000). Caspase-9: involvement in secondary death of axotomized rat retinal ganglion cells in vivo.. Brain Res Mol Brain Res.

[5] Kermer P, Klocker N, Labes M, Thomsen S, Srinivasan A, Bahr M (1999). Activation of caspase-3 in axotomized rat retinal ganglion cells in vivo.. FEBS Lett.

[6] Barde YA (1989). Trophic factors and neuronal survival.. Neuron.

[7] Munemasa Y, Kim SH, Ahn JH, Kwong JM, Caprioli J, Piri N (2008). Protective effect of thioredoxins 1 and 2 in retinal ganglion cells after optic nerve transection and oxidative stress.. Invest Ophthalmol Vis Sci.

[8] Isenmann S, Wahl C, Krajewski S, Reed JC, Bahr M (1997). Up-regulation of Bax protein in degenerating retinal ganglion cells precedes apoptotic cell death after optic nerve lesion in the rat.. Eur J Neurosci.

[9] McKernan DP, Cotter TG (2007). A Critical role for Bim in retinal ganglion cell death.. J Neurochem.

[10] Peng PH, Chiou LF, Chao HM, Lin S, Chen CF, Liu JH, Ko ML (2010). Effects of epigallocatechin-3-gallate on rat retinal ganglion cells after optic nerve axotomy.. Exp Eye Res.

[11] Luo JM, Cen LP, Zhang XM, Chiang SW, Huang Y, Lin D, Fan YM, van Rooijen N, Lam DS, Pang CP, Cui Q (2007). PI3K/akt, JAK/STAT and MEK/ERK pathway inhibition protects retinal ganglion cells via different mechanisms after optic nerve injury.. Eur J Neurosci.

[12] Koeberle PD, Ball AK (1999). Nitric oxide synthase inhibition delays axonal degeneration and promotes the survival of axotomized retinal ganglion cells.. Exp Neurol.

[13] Baur JA, Sinclair DA (2006). Therapeutic potential of resveratrol: the in vivo evidence.. Nat Rev Drug Discov.

[14] Sun AY, Wang Q, Simonyi A, Sun GY (2010). Resveratrol as a therapeutic agent for neurodegenerative diseases.. Mol Neurobiol.

[15] Pallàs M, Casadesús G, Smith MA, Coto-Montes A, Pelegri C, Vilaplana J, Camins A (2009). Resveratrol and neurodegenerative diseases: activation of SIRT1 as the potential pathway towards neuroprotection.. Curr Neurovasc Res.

[16] Howitz KT, Bitterman KJ, Cohen HY, Lamming DW, Lavu S, Wood JG, Zipkin RE, Chung P, Kisielewski A, Zhang LL, Scherer B, Sinclair DA (2003). Small molecule activators of sirtulins extend Saccharomyces cerevisiae lifespan.. Nature.

[17] Wood JG, Rogina B, Lavu S, Howitz K, Helfand SL, Tatar M, Sinclair D (2004). Sirtuin activators mimic caloric restriction and delay ageing in metazoans.. Nature.

[18] Brooks CL, Gu W (2009). How does SIRT1 affect metabolism, senescence and cancer?. Nat Rev Cancer.

[19] Parker JA, Arango M, Abderrahmane S, Lambert E, Tourette C, Catoire H, Neri C (2005). Resveratrol rescues mutant polyglutamine cytotoxicity in nematode and mammalian neurons.. Nat Genet.

[20] Araki T, Sasaki Y, Milbrandt J (2004). Increased nuclear NAD biosynthesis and SIRT1 activation prevent axonal degeneration.. Science.

[21] Sönmez U, Somez A, Erbil G, Tekmen I, Baykara B (2007). Neuroprotective effects of resveratrol against traumatic brain injury in immature rats.. Neurosci Lett.

[22] Della-Morte D, Dave KR, DeFazio RA, Bao YC, Raval AP, Perez-Pinzon MA (2009). Resveratrol pretreatment protects rat brain from cerebral ischemic damage via a sirtulin 1-uncoupling protein 2 pathway.. Neuroscience.

[23] Kim SH, Munemasa Y, Kwong JM, Ahn JH, Mareninov S, Gordon LK, Caprioli J, Piri N (2008). Activation of autophagy in retinal ganglion cells.. J Neurosci Res.

[24] Shindler KS, Ventura E, Rex TS, Elliott P, Rostami A (2007). SIRT1 activation confers neuroprotection in experimental optic neuritis.. Invest Ophthalmol Vis Sci.

[25] Sha O, Kwong WH (2006–07). Postnatal developmental changes of vitreous and lens volumes in Sprague-Dawley rats.. Neuroembryol Aging.

[26] Van Bergen NJ, Wood JP, Chidlow G, Trounce IA, Casson RJ, Ju WK, Weinreb RN, Crowston JG (2009). Recharacterization of the RGC-5 retinal ganglion cell line.. Invest Ophthalmol Vis Sci.

[27] Siemann EH, Creasy LL (1992). Concentration of the phytoalexin resveratrol in wine.. Am. J. Eno. Vatic..

[28] Barger JL, Kayo T, Vann JM, Arias EB, Wang J, Hacker TA, Wang Y, Raederstorff D, Morrow JD, Leeuwenburgh C, Allison DB, Saupe KW, Cartee GD, Weindruch R, Prolla TA (2008). A low dose of dietary resveratrol partially mimics caloric restriction and retards aging parameters in mice.. PLoS ONE.

[29] Marambaud P, Zhao H, Davies P (2005). Resveratrol promotes clearance of Alzheimer's disease amyloid-beta peptides.. Biol Chem.

[30] Frassetto LJ, Schlieve CR, Lieven CJ, Utter AA, Jones MV, Agarwal N, Levin LA (2006). Kinase-dependent differentiation of a retinal ganglion cell precursor.. Invest Ophthalmol Vis Sci.

[31] Jaliffa C, Ameqrane I, Dansault A, Leemput J, Vieira V, Lacassagne E, Provost A, Bigot K, Masson C, Menasche M, Abitbol M (2009). Sirt1 Involvement in rd10 Mouse Retinal Degeneration.. Invest Ophthalmol Vis Sci.

[32] Wenzel E, Solodo T, Erbersdobler H, Somoza V (2005). Bioactivity and metabolism of trans-resveratrol orally administered to Wistar rats.. Mol Nutr Food Res.

[33] Luo J, Nikolaev AY, Imai S, Chen D, Su F, Shiloh A, Guarente L, Gu W (2001). Negative control of p53 by Sir2α promotes cell survival under stress.. Cell.

[34] Vaziri H, Dessain SK, Ng-Eaton E, Imai SI, Frye RA, Pandita TK, Guarente L, Weinberg RA (2001). hSIR2(SIRT1) functions as an NAD-dependent p53 deacetylase.. Cell.

[35] Kops GJ, Dansen TB, Polderman PE, Saarloos I, Wirtz KW, Coffer PJ, Huang TT, Bos JL, Medema RH, Burgering BM (2002). Forkhead transcription factor FOXO3a protects quiescent cells from oxidative stress.. Nature.

[36] Kobayashi Y, Furukawa-Hibi Y, Chen C, Horio Y, Isobe K, Ikeda K, Motoyama N (2005). SIRT1 is critical regulator of FOXO-mediated transcription in response to oxidative stress.. Int J Mol Med.

[37] Chen J, Zhou Y, Mueller-Steiner S, Chen LF, Kwon H, Yi S, Mucke L, Gan L (2005). SIRT1 protects against microglia-dependent amyloid-beta toxicity through inhibiting NF-kappaB signaling.. J Biol Chem.

[38] Cohen HY, Lavu S, Bitterman KJ, Hekking B, Imahiyerobo TA, Miller C, Frye R, Ploegh H, Kessler BM, Sinclair DA (2004). Acetylation of the C-terminus of Ku70 by CBP and PCAF controls Bax-mediated apoptosis.. Mol Cell.

[39] Walle T, Hsieh F, DeLegge MH, Oatis JE, Walle UK (2004). High absorption but very low bioavailability of oral resveratrol in humans.. Drug Metab Dispos.

[40] Li C, Wang L, Huang K, Zheng L (2012). Endoplasmic reticulum stress in retinal vascular degeneration: protective role of resveratrol.. Invest Ophthalmol Vis Sci.

